# Supplementary material for: Perceived work-related stress and associated factors among the surgical workforce in a Nigerian tertiary health facility: A cross-sectional study
Source: PLOS Glob Public Health. 2024 Nov 18;4(11):e0003959. doi: 10.1371/journal.pgph.0003959 (PMC11573183; doi:10.1371/journal.pgph.0003959)
Supplement: S1 File — (PDF) [file pgph.0003959.s001.pdf]

APPENDIX I

QUESTIONNAIRE

Dear Respondent,

This research questionnaire is designed to assess Prevalence and determinants of perceived work-related stress among surgical team members in Ahmadu Bello University Teaching Hospital, Shika Zaria, Kaduna State, Nigeria. Kindly provide accurate and up to date information to contribute meaningfully to the study. Information provided will be used for research purposes and treated with utmost anonymity and confidentiality.

If you agree to participate in the study, kindly sign the consent column below.

Sign …………………………..

Thank you for your anticipated cooperation.

Section One: Socio-demographic data

1 What is your gender? Male ( ) Female ( )

2. What is your age in years? ______________________

3P.Tribe?_______________________________

4. Religion? Islam ( ) Christianity ( ) others (specify)___________________________

5. Years of experience in the operating theatre? _______________________

6. What is your Occupation (cadre)? Doctor ( ) Nurse ( )

7. Highest level of education? PhD ( ) Fellowship ( ) Masters/MSc ( ) PGD ( ) BSc. ( ) HND/HND Equivalent ( ) Diploma ( ) others (specify) ______________________________

8. Marital Status: Single ( ) Married ( ) Separated ( ) Divorcee ( ) Widow ( )

9. If married, partner living together ( ) Partner living separately ( )

10. Number of your dependents? _____________________________________

11. Average monthly income #____________________________________

12. Average working hours per week __________________________________

13. Your Specialty as a surgical team member? Consultants surgeon ( ) Consultant Anaesthesiologist ( ) Surgical trainee ( ) Anaesthesiologists trainees ( ) Perioperative Nurse ( ) Nurse Anaesthetists ( )

14. Theatres you are currently working in. (You can tick more than one option) Modular ( )

A & E ( ) D/S ( ) Ophthalmic ( ) Day Care ( ) All ( )

Section Two: Prevalence and perceived work-related stress

The questions in this scale ask you about your feelings and thoughts about work-related stress. In each question, you are required to indicate by ticking, how often you felt or thought about work-related stress in ABUTH theatres

15. How stressful do you perceive your job in the operating theatre? Low stress ( ) Moderate stress ( ) Severe stress ( ) lack of stress (   )

**S/N**	**Questions**	**Never**	**Almost Never**	**Sometimes**	**Fairly Often**	**Very Often**
16	How often have you been upset because of something that happened unexpectedly?					
11	How often have you felt that you were unable to control the important things in your life?					
18	How often have you felt nervous and "stressed"?					
19	How often have you felt confident about your ability to handle your personal problems?					
20	How often have you felt that things were going your way?					
21	How often have you been able to control irritations in your life?					
22	How often have you felt that you were on top of things?					
23	How often have you found that you could not cope with all the things that you had to do?					
24	How often have you been angered because of things that were outside of your control?					
25	How often have you felt difficulties were piling up so high that you could not overcome them?					

26. **How often do you perceived your exposure to these categories of work-related stress in the operating theatre?**

**Categories of work-related stress**	**Never**	**Almost never**	**Sometimes**	**Fairly often**	**Very often**
Working environment					
Social/Interpersonal					
Psychological					
Physical					
Organizational/institutional					

28. Have you ever exposure to any form of hazard in the operating room before? Yes (   ) No (   ) Unsure (   )

Section Three: Perceived causes of work related stress in the operating theatre

Please indicate to what extent you agreed or disagreed with the following work-related factors

**SN**	**Job related factors**	**SA**	**A**	**N**	**D**	**SD**
29	Lack of support from seniors					
30	Negative experience with patients/relatives					
31	Complexity of your work					
32	Conflicting job demands (cognitive)					
33	Workload					
34	Role conflict					
35	lack of balance between personal and work life					
36	Role ambiguity					
37	Dealing with death and dying					
38	Inadequate preparations of patients for surgery					

SN	Organizational/institutional related factors	SA	A	N	D	SD
39	Job policy and procedure					
40	Lack of promotion					
41	Lack of availability of supplies and equipment					

SN	Interpersonal related factors	SA	A	N	D	SD
42	Lack of peer support					
43	Lack of Supervisor's support					
44	Communication problems					
45	lack of balance between personal and work life					

SN	Physical/Working environment related factors	SA	A	N	D	SD
46	Poor housekeeping of the theatre					
47	Lack of equipment to work with					
48	Space for work					
49	Safety of your personal belongings					
50	The level of noise in the theatre					
51	The level of lighting in the theatre					
52	The temperature (cooling system) of the theatre					
53	Risk for injury					
54	Exposure to dangerous chemicals					
55	The nature of the convenience					

56	The nature of the changing room					
57	Your safety in the theatre					

Section four: Perceived outcome of work-related stress

Please indicate to what extent you agreed or disagreed with following perceived outcome

**SN**	**Outcomes**	**SA**	**A**	**N**	**D**	**SD**
58	Interpersonal conflict					
59	Poor performance					
60	Low morale/Motivation					
61	Poor job satisfaction					
62	Poor client/patient outcome					
63	Sleeping disorders					
64	Increase medical errors					
65	Increased health workers turnover					
66	Job absenteeism					
67	Musculoskeletal pains and fatigue					
68	High blood pressure					
69	Depression					
70	Anxiety					
