# Supplementary material for: Perceived work-related stress and associated factors among the surgical workforce in a Nigerian tertiary health facility: A cross-sectional study
Source: PLOS Glob Public Health. 2024 Nov 18;4(11):e0003959. doi: 10.1371/journal.pgph.0003959 (PMC11573183; doi:10.1371/journal.pgph.0003959)
Supplement: Attachment — Submitted filename: Review Plos GH.pdf [file pgph.0003959.s004.pdf]

Dear Authors,

Thank you for sharing a revised version of your work titled "Perceived work-related stress and associated factors among the surgical workforce in a Nigerian tertiary health facility: A cross-sectional study." I thoroughly enjoyed reading it. Please find some comments for the text shared herewith:

Introduction

Line 75 page "I" needs to be in small case

Line 78 add comma between "low motivation workplace bullying"

Results

Please describe why tribe was selected as a variable by the authors, and how it relates in its significance as a stressor in the context of this study population.

Discussion

Line298 to 299, I believe the sentence is supposed to say "Conversely, many of the surgical workforce might have not been *comfortable* filling in the survey due to fear of repression by the hospital."

In the discussion section, add numerical data from the studies cited to show the comparison between the present study and previous evidence.

Line 335, rephrase from "that nurses who had less age" to younger nurses

Recommendations

The recommendations section has a lot of overflow of points from the discussion. Addtionally, the recommendations are very generalized and need to be further tailored to the study populations.

A suggestion for the authors is to identify perhaps 3 main recommendations and share brief details on their execution. Include specific, actionable interventions that would help enhance the applicability of the recommendation to the study setting.

Conclusion

The authors are recommended to look further into smart experiments in how to reduce workload stress. It is worthwhile to discuss that normative measures of reducing health workforce stressors (more training, workplace social hours) go so far if there are not targeted interventions on improving workflow, work hours, interpersonal communication chains etc.

Regards,

Hamaiyal Sana, MBBS.
