# Supplementary material for: Perceived work-related stress and associated factors among the surgical workforce in a Nigerian tertiary health facility: A cross-sectional study
Source: PLOS Glob Public Health. 2024 Nov 18;4(11):e0003959. doi: 10.1371/journal.pgph.0003959 (PMC11573183; doi:10.1371/journal.pgph.0003959)
Supplement: Attachment — Submitted filename: Response to Reviewers.docx [file pgph.0003959.s003.docx]

**16^th^ July 2024**

C/O Society for Family Health,

No. 8 Port Harcourt Crescent Abuja, Nigeria

Editor in Chief,

PLOS Global Public Health

**Point-Point Response to Editor:**

We thank the editors and reviewers for the comments, and we have attached all comments and their responses on each of the places they are directed at. Please do our responses and a track change on the manuscript that responds to them:

Journal Requirements:

1. Please include a complete copy of PLOS’ questionnaire on inclusivity in global research in your revised manuscript. Our policy for research in this area aims to improve transparency in the reporting of research performed outside of researchers’ own country or community. The policy applies to researchers who have travelled to a different country to conduct research, research with Indigenous populations or their lands, and research on cultural artefacts. The questionnaire can also be requested at the journal’s discretion for any other submissions, even if these conditions are not met. Please find more information on the policy and a link to download a blank copy of the questionnaire here: <https://journals.plos.org/globalpublichealth/s/best-practices-in-research-reporting>. Please upload a completed version of your questionnaire as Supporting Information when you resubmit your manuscript.

We thank the journal editor for this comment, and this have been addressed and we have completed a copy of PLOS’ questionnaire on inclusivity in global research in our revised manuscript.

2. In this instance it seems there may be acceptable restrictions in place that prevent the public sharing of your minimal data. However, in line with our goal of ensuring long-term data availability to all interested researchers, PLOS’ Data Policy states that authors cannot be the sole named individuals responsible for ensuring data access (<http://journals.plos.org/plosone/s/data-availability#loc-acceptable-data-sharing-methods>). Data requests to a non-author institutional point of contact, such as a data access or ethics committee, helps guarantee long term stability and availability of data. Providing interested researchers with a durable point of contact ensures data will be accessible even if an author changes email addresses, institutions, or becomes unavailable to answer requests.
Before we proceed with your manuscript, please also provide non-author contact information (phone/email/hyperlink) for a data access committee, ethics committee, or other institutional body to which data requests may be sent. If no institutional body is available to respond to requests for your minimal data, please consider if there any institutional representatives who did not collaborate in the study, and are not listed as authors on the manuscript, who would be able to hold the data and respond to external requests for data access? If so, please provide their contact information (i.e., email address). Please also provide details on how you will ensure persistent or long-term data storage and availability.

We thank the editor for this important comment, and we have reflected this in the declaration section of the updated manuscript. We quote as cited here:

***Data Availability: All relevant data will be available with a substantial request to the data repository centre at the Society for Family Health (SFH) to ensure sustainability and long-term availability at*** [***info@sfhnigeria.org***](mailto:info@sfhnigeria.org) ***or directly through the corresponding author at*** [***hwada@sfhnigeria.org***](mailto:hwada@sfhnigeria.org) ***or*** [***hasawa2011@gmail.com***](mailto:hasawa2011@gmail.com)

Additional Editor Comments (if provided):

The subject of research is important and understudied and the methodology is sound. The paper can be greatly improved with the feedback provided by our reviewers. I agree with both reviewers that the manuscript should be reviewed by either a native English speaker before sending the revised version to improve clarity and information flow. I commend the authors for the important work and look forward to reading the revised version.

Reviewers' comments: Reviewer's Responses to Questions

**Comments to the Author**

Reviewer #1: Thank you for allowing me to review this work. I commend the authors on addressing such an important topic. As highlighted in this manuscript, it is crucial to protect the mental health of the health workforce, with work-related stress being an important factor. This issue is particularly pressing in LMICs, where it is still underreported due to stigma and other barriers. Overall, the findings are compelling; however, I recommend substantial revisions, with particular attention to significant rewriting.

We thank the reviewer for finding time to review our manuscript. We have addressed all comments raised herein below and in the updated manuscript using track changes.

Title:
1. Several statements, including the title, imply causation and national impact. “Work-related stress and associated factors among surgical team members in Nigeria’s operating room: A cross-sectional study”
- I suggest adding words such as "Perceived" to mitigate the implication of causation.

We thank the reviewer for this comment, and we have added “perceived” to mitigate the implication of causation. The title now read: “*Perceived work-related stress and associated factors among the surgical workforce in a Nigerian tertiary health facility: A cross-sectional study*”

- Instead of "surgical team members," I suggest using "Surgical Workforce" and maintaining this terminology consistently throughout the text.

We thank the reviewer for this comment, and we have changed surgical team members to surgical workforce. The new title read: “*Perceived work-related stress and associated factors among surgical workforce in a Nigerian tertiary health facility: A cross-sectional study*”
- Be cautious with the term "in Nigeria’s operating room" Since the study is based on a single center with a limited cohort, it cannot be generalized to other facilities in Brazil. Consider rephrasing to reflect this limitation.

We thank the reviewer for this comment, and we have changed the title to read as: “*Perceived work-related stress and associated factors among the surgical workforce in a Nigerian tertiary health facility: A cross-sectional study*”

**Abstract:**
1. I would suggest the authors add numerical results in the abstract for clarity.
We thank the reviewer for this comment. We have added numerical results in the abstract.
2. Include the time period during which the study was conducted to provide context.
We thank the reviewer for this comment. We have added the time period of the study in the abstract (see line 26-27) We also explained deeper in the method section on what was done when (See line 102-104).
3. Suggested sentence: "The study found an overall prevalence of work-related stress, with 65% of participants reporting moderate levels of stress."

We thank the reviewer for this comment and the suggestion raised. We have restructured the sentence in line with the suggestion made for the abstract (See line 30-32).
4. Clarify the sample size (189 or 177?). Ensure consistency in the reported number of participants.
We thank the reviewer for this comment. We have clarified the sample size in the methodology section and made sure it is consistent (See line 28-29).
**Introduction:**
1. While the introduction appears to be sound, the writing is unclear, making it difficult to follow. I advise the authors to work with a writing coach or copy editor to improve the flow and readability of the text. This also applies to the discussion section.
We thank the reviewer for this comment. We have employed the work of a copywriter/native speaker, who has evaluated the flow, made suggestions, and has been affected in the entire manuscript.
2. There is no need to define the surgical workforce in the introduction as it is now: “The perioperative teams consist of several kinds of professionals, including surgeons, anesthesiologists, nurses, and surgical technicians [18]. Nurses working in the operating room include scrub and circulating nurses, as well as nurse anesthetists [19].” In any case, this should be moved to the methodology section to define what was considered by the authors as an inclusion criterion for this study.
We thank the reviewer for this comment. We have removed the definition as suggested. We also have added rationale for selecting only surgical specialties and patient volume as it helps the readers to understand the specific relevance and implications of our study.
Methods:
I commend taking into consideration different specialties and different workforces, as well as using standardized text and rigorous methodology. However, there are some points to be clarified:

1. Please cite the “National Institute of Occupational Safety and Health (NIOSH) Generic Job Stress Questionnaire.”
We thank the reviewer for this comment. We have inputted the citation in the manuscript (Page 7 and line 167) and we have also updated the reference section to reflect this on the reference list: No 33.
2. Is the survey available? This is important for reproducibility and to further understand how the questionnaire was developed, and the questions asked.
We thank the reviewer for this comment. We have attached the final questionnaire as a supplement to this research which can be accessible to the public on open access (please see the supplementary list as an appendix for your perusal).
3. It would be helpful to provide a brief background on the country’s surgical training, surgical work process, and workload, either in the “Study Setting and Design” section or in the discussion. Expand on the explanation of this hospital’s structure/types and compare it to other hospitals in Nigeria. Does it have a higher volume? Higher work hours? Less staff? What are the characteristics of this hospital and setting? This would help in understanding the results and their association with work-related stress.
We thank the reviewer for this comment. We have updated this section on the discussion part of the manuscript (see line XXX).
4. Can the authors elaborate on the selection bias of their study sample? This is briefly acknowledged but not discussed in detail in the limitations. For example, how might the survey length (many questions), the topic (mental health, work-related stress), and other commitments (as work tasks and work volume are important issues) influence the results?
We thank the reviewer for this comment. We have updated this section on the limitation part of the manuscript.
5. Could the authors expand on how this test was performed and how the questions were modified? Based on what? Was it through cognitive tests, pilot tests, or focus groups? Did respondents share what they understood or not? How was the process documented and what needed to change? “The survey underwent a pre-test on 10% of the sample, drawn from a similar group in a different institution (Barau Dikko Specialist Hospital, Kaduna). Based on the preliminary findings, minor adjustments were made to enhance the survey.”
We thank the reviewer for this comment. We have highlighted in full, how we did the pilot testing to establish the validity (content and face) and reliability using Cronbach’s alpha for internal consistency and stability of the instrument (see lines 178-192).
6. I would suggest the authors expand on the process “A descriptive cross-sectional study was conducted from January 2021 to January 2022 within the operating theatres of Ahmadu Bello University Teaching Hospital (ABUTH) in Zaria, Nigeria.” Who delivered the surveys? Were they digital or paper-based? How frequently were they administered? How much time did it take to complete each survey? Was it before or after the surgical procedures? Before or after the staff shift? It would be important to understand more.
We thank the reviewer for this comment. We have updated this section and highlighted how this was done in the data collection section as suggested (see lines 196-205).
Results:
1. The results in the table do not sum up correctly:
177 for gender, 178 for age, and 175 for cadre.

We thank the reviewer for this comment. The data collected had errors on 1-2 questionnaires in the reported table with -1/2 responses as it wasn’t filled. However we have traced back the allocation of the questionnaire to have a true picture of what cadre and age they represented and have been affected in the updated table (see table 2).
2. Please standardize how numbers are reported.
For instance “The majority of participants (n=115, or 65per cent) reported moderate levels of work-related 214 stress, whereas 20.3 per cent reported severe and 11.9 per cent reported low levels, 215 respectively” then “The majority of participants were physicians (n=116; 66.3%), and males constituted 59.9% (n=106) of the sample. More than half of the participants, accounting for 53.7% (n=95), identified as Muslims. The ethnic distribution reflected Hausa (48.2%), Yoruba (14.1%), and Igbo (11.3%)
We thank the reviewer for this comment. We have standardized reportage to a 1 decimal place reportage (see updated result section).
3. As different specialties were collected, it would be interesting to have the distribution reported. Have the authors assessed the difference in stress burden among these specialties?
We thank the reviewer for this comment. We avoided disaggregating the result of different specialties as it was discussed following the pilot study due to the nature of controversies it might generate and also might not provide the true stress nature of those specialties. We have taken a new measure to acknowledge this in our limitation section and recommend that a more robust study should be able to delve into the truer nature of stress in those specialties (see updated limitation section).
4. What was the completion rate of the survey? How were incomplete responses handled?
We thank the reviewer for this comment. We have acknowledged the completion rate of the study in our abstract and result section:

Abstract: Employing a cross-sectional descriptive design and a stratified, proportionate sampling technique, 189 surgical team members were recruited, with only 177 personnel fully completing the survey process (93.7% completion rate).

Result: … The result following the data collection shows that out of 189 surgical team members that were recruited for this study, only 177 personnel fully completed the survey process (93.7% completion rate). (see abstract line 29-34 and updated result section on line 238-241).
5. The terms "conflicting job demands (cognitive)" and "Organizational factors, such as job policy and procedure" are unclear, please clarify what they mean. Overall the categories could benefit from more detail on what they encompass and how they were explained to respondents.

Discussion/Limitations:
The discussion brings many important insights, reflecting on the manuscript's findings and comparing them with existing literature. However, I would like to bring up some points that can improve this section:

1. Can the authors expand on what could be the reasons behind their findings? For instance, on the predictors of perceived work-related stress among surgical team members.

We thank the reviewer for this comment. We reviewed the method and discussion section on the predictors and highlighted the reason behind them (see line 338).
2. Enhance the clarity of your sentences and paragraphs by breaking them down into simpler, more concise statements.
We thank the reviewer for this comment. We have employed a native copywriter and broken the complex sentences for clarity.
3. The sentence “A vital recommendation for policymakers is ensuring that there is an increased training for human resources for health in surgical work, reduced workload and having a define time role, particularly for residents as Nigeria define its human resource for health policy framework in the Nigeria Health Sectoral Renewal Investment Initiative.” is a clear example of how it would benefit from breaking it down. Additionally, Please expand on this Policy. How many hours are recommended? By how much does Nigeria usually comply? What are the penalties of not complying to this law?
We thank the reviewer for this comment. We have expanded this on the discussion section and highlighted all that have been raised (see new recommendation section with adequate references).
4. On this sentence “This contrasts with the findings that showed overall low levels of perceived work-related stress, suggesting a potential steady increase in stress levels in the operating theatre” Authors must be cautious, as it is not appropriate to infer an increase in work-related stress across the country based on a single report from another hospital in a different region and time. I suggest removing this statement from the manuscript or revising it to indicate that it would be valuable to assess the situation over time at this center and other hospitals in Nigeria to detect an increase in work-related stress nationwide. Additionally, an important aspect to consider is that this study was conducted in 2021, during which the COVID-19 pandemic significantly impacted the surgical workforce. The ongoing consequences of the backlog generated during the pandemic, which were not mentioned in the discussion of this manuscript, should also be addressed.
We thank the reviewer for this comment. We have removed the statement highlighted and added new caveat on our study limitation related to COVID-19 both on the discussion and limitation section as highlighted (see line 390-395).
5. Please be clear when describing findings from your study and other studies. When referencing other studies, provide specific information about their origin. For example, instead of writing: “Multiple regression analysis is consistent with a study that identifies professional cadre, age, and tribe as significant predictors of perceived work-related stress among surgical team members [45]. They also reported a significant relationship between age, organizational-related stressors, marital status, and educational qualification with work-related stress [45].”
We thank the reviewer for this comment. We have considered the suggestion and adapted as advised.
Consider writing: “Our multiple regression analysis identified professional cadre, age, and tribe as significant predictors of perceived work-related stress. These findings are compatible with similar studies assessing the surgical workforce. For instance, a study in Malawi identified a relationship between sources of stress and demographic variables in nurses. Another study from [location] found [specific findings]. Interestingly, a study from [location] found [other specific findings].”
We thank the reviewer for this comment. We have considered the suggestion and adapted as advised.
Ensure similar clarity throughout the text. For example: “The associated stressors were found to be age group, professional cadre, and tribe, which can have negative consequences on the quality of caregiving [49].”
Should be clarified to: “Our study found that age group, professional cadre, and tribe are associated stressors, which can negatively impact the quality of caregiving. This is consistent with findings from a study in [location], which also highlighted these factors as significant stressors [49].”
We thank the reviewer for this comment. We have considered the suggestion and adapted as advised (see line 375-379).
6. Can the authors comment on whether there may be an over- or under-estimate of work-related stress based on their survey? In reality, there may be bias due to self-selection of individuals filling in the survey because they are subject to work-related stress and may feel empowered to fill in the survey, whereas those who are not subject to work-related stress may not feel the need to fill in the survey. Conversely, professionals subject to work-related stress may be uncomfortable filling in the survey, for example, due to fear of repression by the hospital.

We thank the reviewer for this comment. We have added in the limitation section that it is a single—center study and showed the implication of it on the generalizability to other settings (see line 308-318 and line 370).
7. It should be acknowledged in the limitations section that this is a single-center study. While it highlights a significant and pressing issue that requires attention and can inform policymakers in enacting change, the findings may not be generalizable to other settings. Therefore, further studies in various hospitals across Nigeria are necessary to gain a comprehensive understanding of the national scenario.
We thank the reviewer for this comment. We have added in the limitation section that it is a single—center study and showed the implication of it on the generalizability to other settings (see line 406-409).
Conclusion:
1. Where is the conclusion of this article? The manuscript should include a clear and concise conclusion that summarizes the main findings, their implications, and potential future research directions.

We thank the reviewer for this comment. We have added the conclusion section of the study as it was an oversight which was cut-off on the submission of the initial draft.

Reviewer #2:

Dear Authors, Thank you for sharing your work titled “Work-related stress and associated factors among surgical team members in Nigeria’s operating room: A cross-sectional study.” The article focuses on a pertinent issue faced by health workers. The study methods are sound and align well with the conclusions drawn from the findings. Here are some recommendations for improving your manuscript:

Introduction:
The introduction has redundancies in the description of the problem. Streamlining this section will enhance readability and focus.

We thank the reviewer for this comment. We have streamlined the section and removed some paragraphs as suggested by reviewer 1. We also have improved it’s readability and focus as highlighted (See Introduction Section).
Further describe the rationale for selecting only surgical specialties. This will help readers understand the specific relevance and implications of the study.

We thank the reviewer for this comment. We have added the reason for selecting only surgical specialty as it increases the flow and readability in both introduction (see line 85-90 and also in the discussion section)
Discuss any potential biases in your sampling technique to provide a balanced view of the study’s strengths and limitations.

We thank the reviewer for this comment. We have added detailed info on our sampling technique in the methods section and we have provided a balanced view of the strengths and limitation in the section (See lines 365-372).
Add a description of the patient volume in the surgical department. This information will better capture the current workload of the study participants and contextualize the stress levels reported.
We thank the reviewer for this comment. We have added a description of patient volume in the institution (See lines 120-123). However, we couldn’t find any literature to reference as it was informal information from the department, the least patient flow information on the internet we could get is in 1970-2000 and not disaggregated (see <https://pubmed.ncbi.nlm.nih.gov/19761173/>)

Methods
The inclusion and exclusion criteria should be justified. Explain why a minimum of six months of experience is necessary and why locum theatre employees and students are excluded.

We thank the reviewer for this comment. We have highlighted in full, how excluded them and the minimum of six months of experience (see the inclusion and criteria section on lines 131-143).
Detail the validation process of the survey instrument, including any pilot testing conducted to ensure reliability and validity.

We thank the reviewer for this comment, which was equally highlighted by another reviewer. We have highlighted in full, how we did the pilot testing to establish the validity (content and face) and reliability using Cronbach’s alpha for internal consistency and stability of the instrument (see lines 178-192).
Elaborate on the statistical tests used and their appropriateness for the data. Provide more context on the use of multiple regression analysis and how it enhances the robustness of your findings.

We thank the reviewer for this comment. We have provided more context for the appropriateness of the multiple regression method .

Data Stratification
Further stratify the data to report specific stressors associated with variables such as gender. This will help shape recommendations tailored to the target population and make the most of the study population.

We thank the reviewer for this comment. We have stratified cadre and other variables, but we avoided disaggregating/stratifying the result of different specialties and genders as it was discussed following the pilot study due to the nature of controversies it might generate and also might not provide the true stress nature of those specialties or gender in the study (males are dominant in our study). We have taken a new measure to acknowledge this in our limitation section and recommend that a more robust study should be able to delve into the truer nature of stress in those specialties or genders (see updated limitation section).

Recommendations and Conclusions
Add a dedicated section for study recommendations and conclusions to summarize your findings. This will provide clear, actionable insights for readers.
Identify specific examples, if available, of successful efforts to mitigate health workers' stressors. This will guide readers on potential solutions and practical applications of your research.

We thank the reviewer for this comment. We have added a dedicated section for study recommendation (see line 338-363), limitation (line 364-375) and conclusions (Line 375-380) to summarize our findings (see updated sections).

Language and Grammar
The manuscript should be reviewed by a native English speaker to address minor grammatical and punctuation errors. This will improve the overall readability and professionalism of your paper.

 We thank the reviewer for this comment. The newly updated manuscript has undergone a comprehensive edit by a native speaker and now reflects a good flow. We also have tracked changes which can be seen by the reviewer as highlighted by another reviewer. We thank him sincerely for this and has improved the readability and professionalism of the paper.
While revising your submission, please upload your figure files to the Preflight Analysis and Conversion Engine (PACE) digital diagnostic tool, <https://pacev2.apexcovantage.com/>. PACE helps ensure that figures meet PLOS requirements. To use PACE, you must first register as a user. Registration is free. Then, login and navigate to the UPLOAD tab, where you will find detailed instructions on how to use the tool. If you encounter any issues or have any questions when using PACE, please email PLOS at figures@plos.org. Please note that Supporting Information files do not need this step.
