# Supplementary material for: Perceived work-related stress and associated factors among the surgical workforce in a Nigerian tertiary health facility: A cross-sectional study
Source: PLOS Glob Public Health. 2024 Nov 18;4(11):e0003959. doi: 10.1371/journal.pgph.0003959 (PMC11573183; doi:10.1371/journal.pgph.0003959)
Supplement: Attachment — Submitted filename: Response to Reviewers.docx [file pgph.0003959.s005.docx]

**14^th^ October 2024**

C/O Society for Family Health,

No. 8 Port Harcourt Crescent Abuja, Nigeria

Editor in Chief,

PLOS Global Public Health

**Point-Point Response to Editor:**

We thank the editors and reviewers for the comments, and we have attached all comments and their responses on each of the places they are directed at. Please do our responses and a track change on the manuscript that responds to them:

Dear Authors,
Please review your reference list to ensure that it is complete and correct. If you have cited papers that have been retracted, please include the rationale for doing so in the manuscript text or remove these references and replace them with relevant current references. Any changes to the reference list should be mentioned in the rebuttal letter that accompanies your revised manuscript. If you need to cite a retracted article, indicate the article’s retracted status in the References list and also include a citation and full reference for the retraction notice.

We thank the journal editor for this comment and noted.

Additional Editor Comments (if provided):

Thank you for the changes made to the manuscript. There is a few small changes that we would like to recommend to strengthen the article. Thank you for your patience during the reviewing process

We thank the journal editor for this comment.

Reviewers' comments:

Reviewer's Responses to Questions

Reviewer #1: I would like to acknowledge and congratulate the efforts that the authors went to, in addressing all of the Reviewer's comments, mine included. The intent and impact of the paper has been significantly improved. I believe this manuscript is important and I think other people would benefit from reading this paper in a publication.

We thank the reviewer 1 for finding time to review our manuscript.

Reviewer 2:

Thank you for sharing a revised version of your work titled “Perceived work-related stress and associated factors among the surgical workforce in a Nigerian tertiary health facility: A cross-sectional study.” I thoroughly enjoyed reading it. Please find some comments for the text shared herewith:

We thank the reviewer for finding time to review our manuscript. We have addressed all comments raised herein below and in the updated manuscript using track changes.

Introduction: Line 75 page “I” needs to be in small case Line 78 add comma between “low motivation workplace bullying”

We thank the reviewer for this comment, and we have effected this change in Line 78.

Results: Please describe why tribe was selected as a variable by the authors, and how it relates in its significance as a stressor in the context of this study population.

We thank the reviewer for this comment, emerging evidence has shown that the majority of professionals who are stressed, try as much to confide with higher colleagues of the same tribe to counteract those feelings, alleviate stress, and create more balance. Studies including Bhui K, (Dinos S, Galant-Miecznikowska M, de Jongh B, Stansfeld S. Perceptions of work stress causes and effective interventions in employees working in public, private and non-governmental organisations: a qualitative study. *BJPsych Bull*. 2016;40(6):318-325. doi:10.1192/pb.bp.115.050823) has shown that perception and personal interventions used outside of work were important to prevent and remedy stress. Therefore, we intended using tribe as a potential stressor to measure as discussed here (Wilson, D. H., German, D., Ricker, A., Gourneau, H., Hanson, G. C., Mayhew, J., Brockie, T. N., & Sarche, M. (2023). Feasibility, acceptability and effectiveness of a culturally informed intervention to decrease stress and promote well-being in reservation-based Native American Head Start teachers. *BMC public health*, *23*(1), 2088. <https://doi.org/10.1186/s12889-023-16913-z>). This has also been highlighted on the study recommendation part.

Discussion: Line 298 to 299, I believe the sentence is supposed to say “Conversely, many of the surgical workforce might have not been comfortable filling in the survey due to fear of repression by the hospital.” In the discussion section, add numerical data from the studies cited to show the comparison between the present study and previous evidence.

We thank the reviewer for this comment, and we have effected this change to reflect what was suggested by the reviewer – changed from uncomfortable to “comfortable”

Line 335, rephrase from “that nurses who had less age” to younger nurses

We thank the reviewer for this comment, and we have effected this change to reflect what was suggested by the reviewer – changed to “younger nurses”

Recommendations: The recommendations section has a lot of overflows of points from the discussion. Additionally, the recommendations are very generalized and need to be further tailored to the study populations. A suggestion for the authors is to identify perhaps 3 main recommendations and share brief details on their execution. Include specific, actionable interventions that would help enhance the applicability of the recommendation to the study setting.

We thank the reviewer for this comment, and we have changed to 3 main recommendations with details of their execution.

Conclusion: The authors are recommended to look further into smart experiments in how to reduce workload stress. It is worthwhile to discuss that normative measures of reducing health workforce stressors (more training, workplace social hours) go so far if there are not targeted interventions on improving workflow, work hours, interpersonal communication chains etc.

We thank the reviewer for this comment, and we have effected this correction as highlighted on lines 412-416
